# Evaluation of the Psychometric Properties of the Digital Health Literacy Instrument (DHLI-BrA) for Use in Brazilian Adolescents

**DOI:** 10.3390/ijerph21111458

**Published:** 2024-10-31

**Authors:** Mariane Carolina Faria Barbosa, Ana Luiza Peres Baldiotti, Ramon Targino Firmino, Saul Martins Paiva, Ana Flávia Granville-Garcia, Fernanda de Morais Ferreira

**Affiliations:** 1 Department of Pediatric Dentistry, Federal University of Minas Gerais (UFMG), Belo Horizonte 31270-901, MG, Brazil; analuizapbaldiotti@hotmail.com (A.L.P.B.); smpaiva@uol.com.br (S.M.P.); femoraisfe@gmail.com (F.d.M.F.); 2 Department of Dentistry, Federal University of Campina Grande (UFCG), Patos 58708-110, PB, Brazil; ramontargino@gmail.com; 3 Postgraduate Program in Dentistry, State University of Paraíba (UEPB), Campina Grande 58429-500, PB, Brazil; anaflaviagg@gmail.com

**Keywords:** health literacy, digital health, adolescent

## Abstract

This study evaluated the psychometric properties of the Portuguese version of Digital Health Literacy Instrument for Brazilian Adolescents (DHLI-BrA). Two hundred and sixty Brazilian adolescents answered the DHLI-BrA and the Brazilian version of quality-of-life and health literacy instruments: WHOQOL-Bref, eHEALS-BrA, NVS-BR, and REALMD-20. Then, they answered a questionnaire on sociodemographics, health, Internet access, and digital health aspects. The data collection was conducted between September and December of 2022.The statistical test assessed internal consistency, stability, discriminant and convergent validities, exploratory factor analysis (EFA), and confirmatory factor analysis (CFA). Item response theory (IRT) analysis using 2PL was applied to performance-based DHLI-BrA. The DHLI-BrA self-reported questions demonstrated almost perfect internal consistency (α and ω = 0.83) and good stability (ICC = 0.906; 95% CI: 0.75–0.95). In EFA and CFA, the best-adjusted model was composed of six factors (χ^2^ = 229.173 (df = 174, *p* = 0.003), CFI = 0.944, TLI = 0.933, RMSEA = 0.035, and RMSR = 0.047). The performance-based DHLI-BrA demonstrated moderate internal consistency (α = 0.57 and KR20 = 0.56) and good stability (ICC = 0.86, 95% CI: 0.76–0.92). In EFA and CFA, the best-adjusted model was composed of a single factor (χ^2^ = 17.901 (df = 14, *p* = 0.2113), CFI = 0.952, TLI = 0.927, RMSEA = 0.033, and RMSR = 0.038). IRT analyses revealed item discrimination ranging from −0.71 to 1.83 and difficulty from −1.53 to 1.02. Convergent validity of the self-reported DHLI-BrA was obtained by its correlation with the eHEALS-BrA (r = 0.45) and REALMD-20 (r = 0.19), besides the performance-based DHLI-BrA with its correlation with the NVS (r = 0.47) and REALMD-20 (r = 0.44). The DHLI-BrA demonstrated adequate psychometric properties to measure the self-reported, performance-based digital health literacy of Brazilian typically developing adolescents.

## 1. Introduction

Technological advances and increased access to the Internet have brought about significant changes to society and transformed people’s lives [1,2]. Digital devices, such as smartphones, notebooks, and tablets, have become healthcare tools with the potential to improve quality of life, disseminate information, and provide access to health services [3,4].

It is estimated that 82.7% of Brazilian households have Internet access, and adolescents are the population group that most access the Internet (90.2%), mainly via smartphones [5]. Therefore, the Internet is part of their lives, and they have a high level of familiarity and ability to use information and communication technologies [4,6,7].

Adolescents have unique characteristics as they undergo significant physical, emotional, and mental maturation, which can lead to questions about their body and health [2,7,8]. Thus, the Internet is an attractive and accessible resource for teenagers to search for information and self-manage their health [4,7,9]. However, the expansion of health information in the media, especially the dissemination of false or misleading content, jeopardizes the decision-making and self-management processes, leading to the development of harmful health beliefs [10,11,12].

It is known that there is an association between high levels of health literacy (HL) and assertive health behaviors in adolescents [13]. Given the high rate of digital insertion in this age group, it is important that adolescents have proper digital health literacy (DHL), so they can search, select, evaluate, interpret, and use health information found online (health 1.0) [14]. They should also have interactive skills such as posting health-related messages on the web and using telehealth applications and services (health 2.0) [15,16]. Digital Health Literacy Instrument (DHLI), developed in the Netherlands in 2017, is an instrument for measuring DHL, including the complete spectrum of eHealth skills (Health 1.0 and Health 2.0) and actual performance-based competencies [15,17]. DHLI has previously been tested for adolescents [4,18] and university students of some countries [19,20,21,22,23], demonstrating an effective mensuration of DHL, low cost, and easy application. This instrument was recently cross-culturally adapted for Brazilian adolescents [17]. However, it is necessary to verify the validity of this instrument for measuring this construct. Thus, this study aimed to evaluate the psychometric properties of the Portuguese version of DHLI-BrA for the public.

## 2. Materials and Methods

### 2.1. Study Design and Population

This is a methodological study evaluating the psychometric properties of the Digital Health Literacy Instrument adapted for Brazilian Adolescents (DHLI-BrA) [17]. It was conducted on a sample of 260 adolescents (aged 13 to 19) enrolled in five public schools in the Brazilian city of Belo Horizonte. The sample size was based on the recommendation of 2 to 20 individuals per instrument item to evaluate the properties [23]; then, considering 10 individuals per item, there is a minimum sample of 210 participants. Given the possibility of possible losses, the sample size was increased by 20% to 252 adolescents. The schools were randomly selected, considering their distribution throughout the municipality and their result in IDEB-2019 (Basic Education Development Index), a teaching quality indicator for Brazilian public schools. Five schools were selected, two among the six schools listed with the best performance in IDEB and three listed among the six as the worst-performing. In addition, the schools were distributed in 5 different locations in the municipality.

### 2.2. Eligibility Criteria

Literate adolescents of both sexes, Brazilian Portuguese native speakers, with access to the Internet, and who were present on the days of data collection were included. Adolescents aged 12 were excluded due to the 1998 Children’s Online Privacy Protection Act, which determines the minimum age of 13 for creating accounts on social networks and using digital services. In addition, adolescents who presented self-reported or school-reported (vision, hearing, or cognitive) problems that impaired their participation were excluded.

### 2.3. Instrument

The Digital Health Literacy Instrument measures digital health literacy. It is a self-reported scale with 21 items that measure the broad spectrum of the eHealth concept, which includes the use of health information on the Internet (health 1.0) and the use of recent applications with interactive technologies (health 2.0). Also, the instrument has 7 items that measure DHL based on the individual’s practical performance [15].

The original instrument is organized into seven skills (factors), with three items each: (1) operational skills—how to use the computer and Internet browsers, (2) navigation skills—how to navigate and find your way around the Web, (3) skills to search for information using correct search strategies, (4) skills to assess information reliability, (5) skills to determine online information relevance, (6) skills to include self-generated content, and (7) skills to protect and respect online privacy [15].

Self-reported items are answered using a 4-point scale (1 to 4), with options ranging from “very easy” to “very difficult” and from “never” to “almost always”, which score is performed invertedly: very easy/never = 4, fairly easy/sometimes = 3, reasonably difficult/often = 2, and very difficult/almost always = 1. The total score is obtained by an average of all responses, with higher scores representing a higher level of DHL. In addition, it is also possible to calculate scores for each of the instrument’s seven skills by averaging the items in each skill [15,17].

The performance-based items have five response options, with only one correct option (score = 1), three incorrect options (score = 0), and one “I don’t know” option (score = 0). The calculation of the total DHL score based on performance is done by adding up the correct answers [15,17]. The full version of the DHLI instrument in Brazilian Portuguese adapted for adolescents is available in the Supplementary Materials of this manuscript.

### 2.4. Data Collection

The data collection with adolescents and their guardians/caregivers was conducted between September and December of 2022. The guardians/caregivers responded to a semi-structured questionnaire about their sociodemographic aspects (age, kinship, education, and income) and about the adolescents’ information (birth order, changes in health, and medication use).

The collection took place at three separate times. Initially, the adolescents were instructed to answer the DHLI-BrA and Brazilian version of the eHealth Literacy Scale for Adolescents (eHEALS-BrA) instruments to measure digital health literacy. They also answered a semi-structured questionnaire to determine their demographic characteristics (gender, age, and education); general health (physical activity and self-assessment of general and oral health); access to the Internet (where the access happens, the main mean of access, use of mobile data, frequency of access, and use of social media); self-assessment of Internet skills; and search behavior for health information online (researched for a doctor/dentist, whether they followed influencers’ recommendations, whether they used the Internet to read/search for health information, appointments scheduling, use of health apps, research for symptoms, and questions to health professionals).

The eHEALS-BrA is a DHL measurement instrument composed of eight self-reported items. The answer options are organized on a 5-point Likert scale (1 to 5), with the total score ranging from 8 to 40 points and higher scores representing higher levels of DHL [14,24].

Secondly, the adolescents responded to the quality-of-life questionnaire, WHOQOL-Bref, and to the health literacy measurement instruments: NVS and REALMD-20. The WHOQOL-Bref is an abbreviated version of the World Health Organization Quality of Life (WHOQOL-100), an instrument for measuring the quality of life recommended by the World Health Organization. It consists of 26 questions with answer options organized on a Likert scale (1 to 5). The instrument covers 4 domains: physical, psychological, social, and environmental [25,26]. Higher scores represent a better quality of life for each instrument’s domain [25,26].

The Newest Vital Sign (NVS-BR) was used to measure HL, an instrument composed of 6 items that assess reading comprehension and numeracy through a simulation with the information on an ice cream label. Each item has a correct answer (1 point), and the total score can vary from 0 to 6 points, in which higher scores represent a better HL [27]. The 20-item Rapid Estimate Adult Literacy in Medicine and Dentistry (REALMD-20) consists of 20 medical and dental terms and assesses the ability to read and pronounce terminologies. Each correctly pronounced word receives 1 point, with higher scores representing a higher HL level [28].

The third moment of data collection happened after two weeks from the DHLI’s first application. Then, 25% of the adolescents were randomly selected to answer the DHLI-BrA instrument again to verify the stability of the instrument. All data collection stages were carried out individually and in a private space within the schools. The instruments were self-administered in printed version; only the NVS and REALDM-20 instruments were applied in an interview format by a single researcher, following the recommended methodologies for each instrument.

### 2.5. Statistical Analysis

Cronbach’s alpha coefficient (α) and McDonald’s omega (ω) were performed as measures of the internal consistency of self-related DHLI-BrA [29,30]. For performance-based items, the reliability was assessed using Cronbach’s alpha coefficient (α) and Kuder–Richardson KR20 [31,32]. Alpha was categorized as follows: 0.81 to 1.0—almost perfect, 0.61–0.80—substantial, 0.41 to 0.60—moderate, 0.21–0.40—reasonable, and 0–0.21—small [29]. The instrument stability was assessed by test–retest reliability after a period of two weeks by using the intraclass correlation coefficient (ICC). The values were categorized as follows: ≤0.40—weak, 0.41–0.60—moderate correlation, 0.61–0.80—good, and 0.81–1.00—excellent [33].

Exploratory factor analysis (EFA) was performed to evaluate the instrument’s dimensionality. The appropriateness of using factor analysis on the data set was assessed using the Kaiser–Meyer–Olkin test (>0.60) and Barlett’s sphericity test (*p* < 0.05). A simple factor solution structure based on reported eigenvalues above 1.0 was used, and the varimax rotation method was performed. The factor loadings were considered: >0.40 acceptable, >0.55 good, >0.63 very good, and >0.71 excellent [34].

Confirmatory factor analysis (CFA) was performed to test the hypothesis based on a theoretical framework and empirical research. The model fitness was evaluated using five indices: the goodness of fit of the chi-square test (χ^2^), the Comparative Fit Index (CFI), the Tucker Lewis index (TLI), the mean square error of approximation (RMSEA), and the standardized root mean square residual (SRMR). For χ^2^, *p*-values > 0.001 indicated a good fit. For the CFI and TLI, values ≥ 0.95 were considered excellent fits, while values between 0.90 and 0.95 indicated an acceptable fit. RMSEA values ≤ 0.05 indicate excellent model fit, while values between 0.05 and 0.08 suggest a good fit and values ≥ 0.10 a poor fit. For the SRMR values, <0.08 indicates a good fit [33,35].

The item response theory (IRT) analysis was conducted in RStudio version 4.2. The analysis was conducted after checking of the dimensionality structure (EFA and CFA). For performance-based items, due to the dichotomous nature of the responses, we used the two-parameter logistic (2PL) IRT model with the mirt package [36]. In this model, two parameters are evaluated. The first is the discrimination (α) parameter, which reflects how strongly the item is related to the latent construct, with values typically ranging from 0 to 3. Higher values indicate a stronger relationship between the item and the latent trait (theta). The second parameter is the difficulty (b) parameter, which indicates the points along the latent trait continuum where each response option has a 50% chance of endorsement. The 2PL model analyzed the parameters of the subjects and items that were shown in an item map. The interpretation of the infit/outfit is influenced by the sample size. In the present study (*n* = 260), values between 0.7 and 1.3 were considered acceptable [37,38].

Construct validity evidence was determined based on discriminant and convergent validity. Convergent and discriminant validity were investigated using the Spearman correlation test with sociodemographic variables (age and average family income), digital health literacy (eHEALS), and health literacy (NVS and REALMD-20). Furthermore, convergent validity was investigated through its correlation with quality of life (WHOQOL-Bref) and by comparing total scores with the variables self-assessment of general health and skills to use the Internet, frequency of Internet access, and behavior in searching for health information and using digital services (Mann–Whitney and Kruskal–Wallis *U* tests, *p* < 0.05).

All statistical tests were performed with SPSS Statistics 21.0 program (SPSS Inc., Chicago, IL, USA), Mplus software (Muthén & Muthén, Version 8.2, Los Angeles, CA, USA), and RStudio Version 4.2.

### 2.6. Ethical Aspects

Prior to this study, the authors of the original instrument were contacted and authorized its implementation [15]. This study was also approved by the Human Research Ethics Committee of the Federal University of Minas Gerais (#CAAE: 58603022.8.0000.5149). In accordance with the recommendations of Resolution 466/2012 of the National Health Council and the Declaration of Helsinki, all participants were informed about the objective of the study and signed an informed consent form (guardians and adolescents over 18 years of age) and an informed assent form (adolescents under 18 years of age).

## 3. Results

Two hundred and sixty adolescents with a mean age of 15.63 (±1.84) participated in this study: one hundred and forty-two (54.6%) were female and one hundred and twelve (45.4%) were male. A total of 139 adolescents (53.7%) attended elementary school II (up to 8 years of schooling), and 122 (48.0%) declared themselves as mixed race. In relation to family aspects, the mother was mostly the family provider (78.5%), and the average family income was BRL $2780.69 (USD $526.64). The average age of parents/tutors was 43.35 years (± 8.14), whose education was equal to or greater than 9 years of formal study (79.1%).

The average self-reported DHLI-BrA total score was 3.02 (±0.37; 2–3.86), and the average administration time was 7.59 min. The 21 self-reported items presented adequate internal consistency based on Cronbach’s alpha = 0.83 and Mc’Donald’s omega = 0.83. Table 1 describes the parameters of the 21 self-reported items from the DHLI-BrA, demonstrating the psychometric quality of the items and indicating that no item should be excluded from the instrument. The parameters presented include the average response value for each item on the scale, the scale variances, the correlation between each item and the total scale score (excluding that specific item), and the Cronbach’s alpha values if that specific item were to be removed.

Test–retest reliability analysis demonstrated excellent reproducibility [ICC = 0.906 (95% CI: 0.75–0.95, *p* < 0.001)]. Preliminary tests demonstrated that the data were adequate to perform an exploratory factor analysis of the 21 self-reported items: KMO = 0.84 and Barlett’s test of sphericity significance (*p* < 0.001).

**Figure 1:**
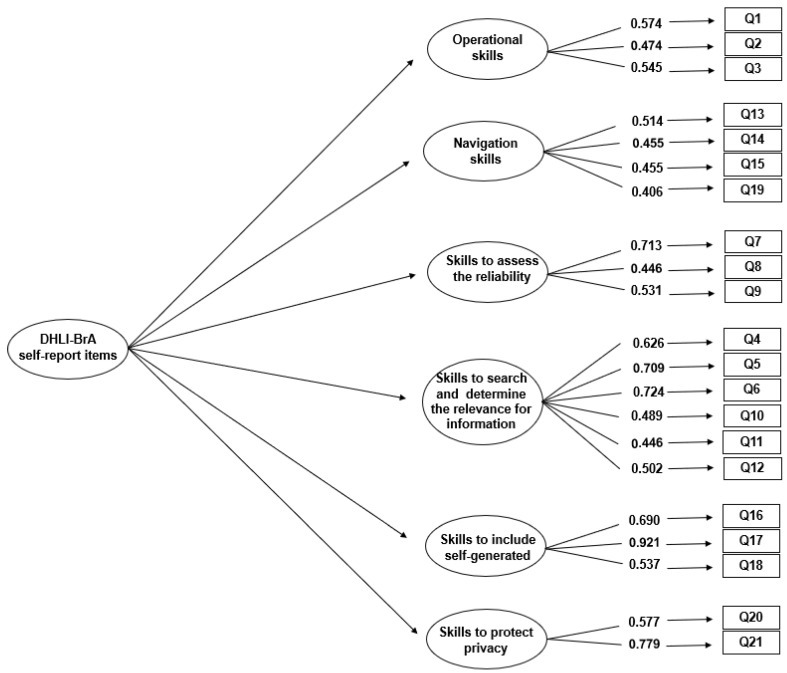
Conceptual representation and factor loadings for the 6-factor solutions of the DHLI-BrA self-reported items.

In the EFA, six factors were found with values ≥ 1.0. However, in the original instrument, seven factors were identified for the 21 self-reported items. As to the theoretical framework, confirmatory factor analysis was conducted for the seven- and six-factor solutions, and the best-adjusted model was the one composed of six factors: χ^2^ = 229.173 (df = 174, *p* = 0.0032), CFI = 0.944, TLI = 0.933, RMSEA = 0.035 (CI: 0.021–0.047), and SRMR = 0.047. The factor loadings were >0.40 (Figure 1). Table 2 presents Cronbach’s alpha for each scale (0.53–0.76) and the correlations between the factors.

For the seven performance-based items, the average total score was 4.15 (±1.72, 0–7), and the average administration time was 10:28 min. Satisfactory reliability was observed (Cronbach’s alpha = 0.57 and KR20 = 0.56), and the detailed parameters of the DHLI-BrA performance-based items are described in Table 3, which demonstrate that all performance-based items should be maintained in the instrument. The assessment of test reliability retesting of the instrument demonstrated adequate reproducibility [ICC = 0.86 (95% CI: 0.76–0.92, *p* < 0.001)]. For the EFA evaluation, it was preliminarily observed that the data were adequate for its performance: KMO = 0.68 and Barlett’s sphericity test significance (*p* < 0.001).

**Figure 2:**
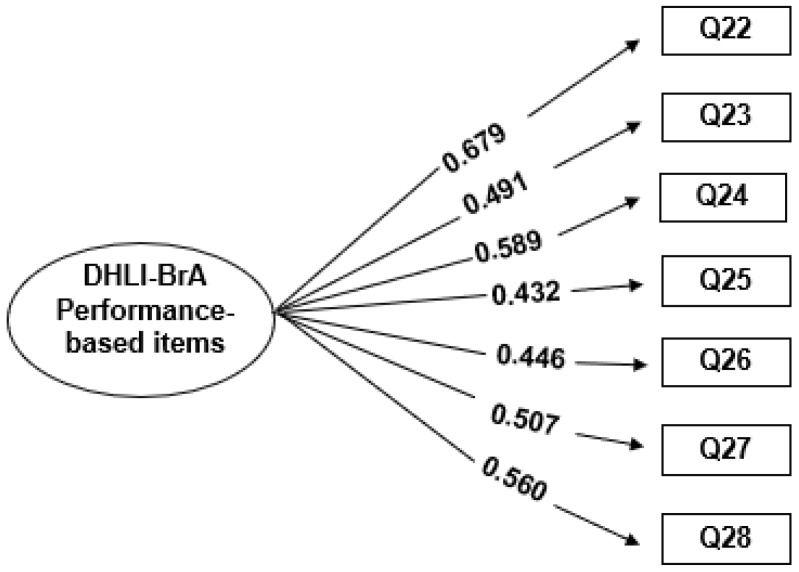
Conceptual representation and factor loadings for the single-factor solution of the DHLI-BrA performance-based items.

In the EFA of the seven performance-based items, two factors were obtained with values ≥1.0. Based on the theoretical framework, confirmatory factor analysis was carried out for the one- and two-factor solutions. However, the two-factor solution presented an unsatisfactory grouping of questions. Then, the one-factor solution was better adjusted, with excellent indices, as demonstrated: χ^2^ = 17.901 (df = 14, *p* = 0.2113), CFI = 0.952, TLI = 0.927, RMSEA = 0.033, and SRMR = 0.038, indicating a good fit of the model. The factor loadings were >0.40 (0.432–0.679) for all instrument items (Figure 2).

In Table 4, we can see the adaptation values concerning the two-parameter logistic model for the seven performance-based items. Only item Q22 had values below the ideal values. In general, the items presented good adaptation values relating to the model, with central values between 0.7 and 1.3 for infit and outfit, verifying the one-dimensionality and confirming the final version of the instrument.

**Figure 3:**
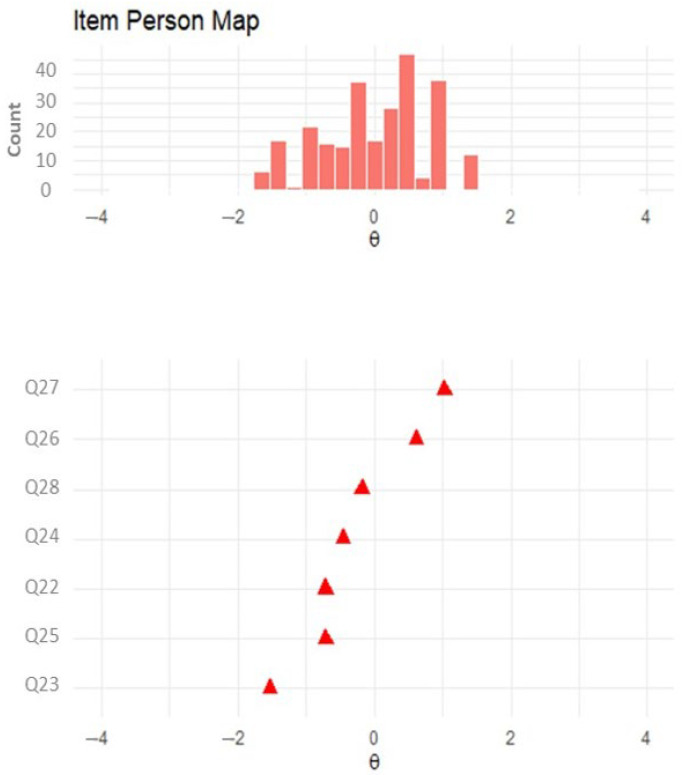
Item map of the final version of the performance-based DHLI-BrA.

Figure 3 shows the skill level of the participants and the difficulty level of the items. It can be seen that most items are located between the −1 and +1 logit points. Items Q23 and Q25 were the easiest (difficulties −1.53 and −0.72 logits, respectively), while items Q26 and Q28 were the most difficult (difficulties 0.61 and 1.02 logits, respectively).

**Figure 4:**
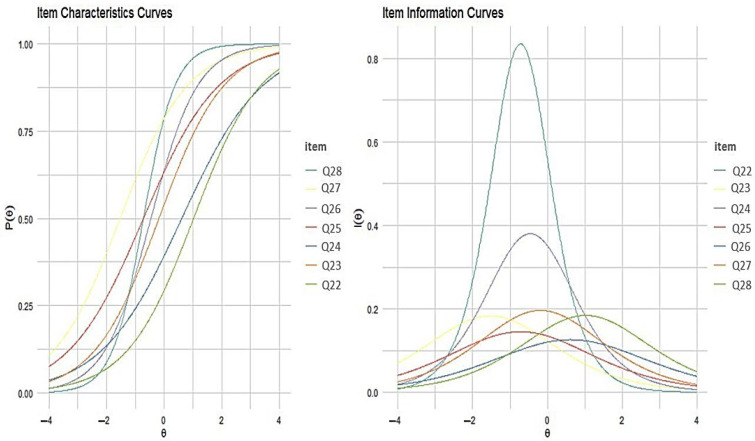
Item response category characteristic curves and item information curves for the performance-based DHLI-BrA.

Figure 4 shows the item characteristic and item information curves plot. Item response category characteristic curves depicted the relationship between the level of performance-based digital health literacy and the probability of selecting the specific option for each item in the scale. The information curves showed that items Q22 and Q24 had the most information, indicating the importance of the response of this item in measuring the level of performance-based DHL.

The discriminant validity of the DHLI-BrA was measured by the correction between the total score and the adolescent’s age, as well as family income. Older adolescents with a higher family income had better self-reported and performance-based digital health literacy (*p* < 0.001) (Table 5).

The convergent validity of the self-reported DHLI-BrA was measured by the significant correlation with the scores of the digital health literacy instrument, eHEALS-BrA (r: 0.192, *p* = 0.002), and health literacy, REALMD-20 (r: 0.192, *p* = 0.002). For the performance-based items, convergent validity was demonstrated by correlation with the health literacy instruments, NVS (r: 0.472, *p* < 0.001) and REALMD-20 (r: 0.445, *p* < 0.001) (Table 5). The convergent validity of the self-reported items was demonstrated by their significant correlation with the perceived quality of life in the physical (r: 0.199, *p* = 0.001), psychological (r: 0.213, *p* = 0.001), and environmental domains (r: 0.183, *p* = 0.033) (Table 5). In addition, the convergent validity was supported by the association with using the Internet to take care of their health, such as searching for professionals, doctors, and dentists (*p* < 0.001) and scheduling appointments online (*p* = 0.014) (Table 6).

The predictive validity of the performance-based DHLI-BrA was demonstrated by the significant association with having a mobile connection to the Internet (*p* = 0.026) and a higher frequency of Internet use (*p* = 0.022) and, also, its association with searching for (*p* = 0.001) and reading (*p* = 0.002) health information online, using health apps (*p* = 0.040), searching for symptoms (*p* = 0.046), and publishing an evaluation/review about medical treatment (*p* < 0.001) (Table 6).

Later, a correlation between the total self-reported DHLI-BrA score and the total score of the performance-based items (r: 0.199, *p* = 0.001) was observed.

## 4. Discussion

This study showed that the 21 self-reported items and the 7 performance-based items of the DHLI-BrA are reliable and valid tools for measuring the DHL of Brazilian adolescents. Therefore, its use can be recommended for epidemiological studies and for health professionals to investigate patients’ individual abilities when using health information on the Internet.

Compared to the original instrument, the DHLI-BrA self-reported items demonstrated similar internal consistency (α = 0.87) and higher levels of test–retest reliability (ICC = 0.77) [15]. As the DHLI was developed recently (2017) [15], it has been adapted and validated for only a few countries and age groups so far [7,17,39,40,41]. Also, during the 2020 coronavirus pandemic, a reduced version of the DHLI was developed to measure DHL regarding COVID-19 in some populations [18,19,20,21,22]. Therefore, this study expands the evidence that the DHLI is an internally consistent and temporally stable measure for measuring DHL.

The original instrument performance-based items presented an internal consistency of α = 0.47, and the authors chose to interpret the items separately [15]. In our study, reliability was slightly higher but still considered moderate (α = 0.57). Based on recent criticism regarding the interpretation of Cronbach’s alpha, we decided to measure the one-dimensionality of the construct, since the small number of items can limit the alpha of an instrument, and making decisions based on a single value can be considered quite simplistic. It is necessary to evaluate other information such as the mean, correlation between items, and variance [42]. We observed that the one-dimensionality of the performance-based items presented satisfactory behavior.

In the analysis of item–total correlations, we noticed that the items that showed a good correlation with the total score had values close to or greater than 0.30. Lower values indicate that an item may not be correlated with the total scale score and should be removed [43]. In this study, self-reported items Q20 and Q21 had a correlation coefficient of 0.11 and 0.15, respectively; however, they were not excluded from the instrument, as the Cronbach’s alpha did not increase with their exclusion. Additionally, for the performance-based items, no outliers were observed.

The average DHLI-BrA score was 3.02 (±0.37), a value similar to those reported in the DHLI validation study for Canadian adults [3.11 (±0.87)] [15], a fact that may be justified by the great familiarity of adolescents with the use of technologies and by it being a phase in which their interest in health information begins [4,7]. For the performance-based items, the average total score was 4.15 (±1.72). It was not possible to compare this result, as this is the first study to measure the one-dimensionality of these items.

The correlation results confirmed the differences in the DHL levels between older adolescents and those with higher family incomes. This fact can be explained by the development and cognitive maturation of adolescents during this phase [8], as well as the development of skills to use the Internet [7] and greater exposure to health information throughout their lives [44]. Thus, family income can influence the health literacy of adolescents [37,38] and access to communication technologies by the population [45].

Functional health literacy, measured by REALMD-20, reflects the basic skills of reading and understanding the terms related to health [28]. In this study, functional HL was found to be moderately correlated with the total score of the performance-based items and weakly correlated with the self-reported items. Similar to the original instrument, we observed a moderate correlation of performance-based items with the total NVS score, which measures health literacy in a more comprehensive and practical way; that is, it assesses the individual’s ability to read, understand, interpret, and make decisions [27]. These results were expected as items based on the DHLI-BrA performance and require practical skills of interpretation and health-related decision-making in the digital environment [15].

Like the original instrument [15], we observed a moderate correlation between the self-reported DHLI-BrA and the eHEALS digital health literacy instrument. As to quality of life, there was a correlation between the best performance on the DHLI-BrA and the physical, psychological, and environment domains.

The better use of the Internet and better self-assessment of the skills to use it were associated with higher levels of digital health literacy. Our results corroborate previous findings in the literature [15,18]. For performance-based items, an association with variables related to eHealth was observed, such as searching and reading health information on the Internet, searching for symptoms, and using health-related applications.

This study provides important guidance on the psychometrics of the DHLI, but it has some limitations. The study involved elementary and high school students in the city of Belo Horizonte, located in Southeastern Brazil. Although schools distributed throughout the city and with different teaching quality classifications (IDEB) were selected, these adolescents may present some characteristics that differ from adolescents from other Brazilian regions. In addition, the digital world is evolving rapidly, and it is likely that some terms, websites, and applications may become outdated within a few years. Therefore, instruments such as DHLI-BrA need to be updated to reflect trends in a timely manner. Finally, we should look at self-reported DHLI items Q20 and Q21 with caution. These items make up the privacy dimension, but their adjustment parameters to the model are not ideal. Therefore, we suggest that future studies seek to validate instruments with better properties to measure this aspect of DHL in adolescents in Brazil.

It is worth highlighting the importance of this study for the safe recommendation of the DHLI-BrA as an instrument for measuring DHL in Brazilian adolescents. From a public health perspective, children and young people constitute a central population group for research and intervention in health literacy [46,47,48]. During youth, fundamental processes of cognitive, physical, and emotional development occur, and health-related behaviors and skills are incorporated [8,48,49]. As health literacy is a variable construct and can be acquired in a lifelong learning process, starting it during adolescence can bring benefits in the short and long term [48].

## 5. Conclusions

The DHLI-BrA demonstrated adequate psychometric properties to measure the self-reported, performance-based digital health literacy of Brazilian adolescents. It is important to highlight that, when using performance-based items, it is necessary to evaluate the main form of Internet access individually, as well as the suitability of the items to keep up with constant technological evolution. In addition, it is interesting that future studies will focus on adapting and evaluating the properties of the instrument for adolescents with disabilities, including those with visual, hearing, and cognitive impairments.

## Figures and Tables

**Table 1:** Scale means, scale variances, item–total correlation, and Cronbach’s alpha if item deleted from the DHLI-BrA self-reported items.

	Scale Mean If Item Deleted	Scale Variance If Item Deleted	Item–Total Correlation	Cronbach’s Alpha If Item Deleted
Q1	59.71	58.2	0.38	0.83
Q2	59.75	58.6	0.30	0.83
Q3	59.88	57.8	0.36	0.83
Q4	60.90	55.3	0.53	0.82
Q5	60.59	53.5	0.61	0.81
Q6	60.89	54.3	0.55	0.82
Q7	61.24	53.8	0.56	0.82
Q8	60.73	56.3	0.30	0.83
Q9	60.35	54.8	0.50	0.82
Q10	60.53	55.6	0.42	0.82
Q11	60.82	55.9	0.39	0.83
Q12	60.87	55.3	0.40	0.83
Q13	60.47	57.7	0.37	0.83
Q14	59.87	57.2	0.34	0.83
Q15	60.96	56.4	0.40	0.83
Q16	60.31	55.0	0.51	0.83
Q17	60.65	54.2	0.47	0.83
Q18	60.38	56.2	0.38	0.83
Q19	60.35	57.5	0.31	0.83
Q20	59.97	60.1	0.11	0.84
Q21	59.79	59.8	0.15	0.83

**Table 2:** Cronbach’s alpha and correlations regarding the factors determined by CFA.

Factors44		Cronbach’s Alpha	Correlations					
			1	2	3	4	5	6
1	Operational skills	0.534	1					
2	Navigation skills	0.528	0.556 *	1				
3	Skills to assess the reliability	0.598	0.620 *	0.841 *	1			
4	Skills to Search and determine the relevance for information	0.757	0.587 *	0.577 *	0.663 *	1		
5	Skills to include self-generated	0.626	0.644 *	0.645 *	0.588 *	0.689 *	1	
6	Skills to protect privacy	0.624	0.175	0.049	0.006	0.453*	0.093	1

* *p* < 0.001.

**Table 3:** Scale means, scale variances, item–total correlation, and Cronbach’s alpha if the item was deleted from the DHLI-BrA performance-based items.

	Scale Mean If Item Deleted	Scale Variance If Item Deleted	Item–Total Correlation	Cronbach’s Alpha If Item Deleted
Q22	3.452	2.197	0.407	0.492
Q23	3.394	2.436	0.255	0.548
Q24	3.544	2.213	0.350	0.512
Q25	3.533	2.372	0.236	0.557
Q26	3.748	2.366	0.235	0.558
Q27	3.617	2.275	0.290	0.537
Q28	3.623	2.460	0.300	0.535

**Table 4:** Two-parameter model for performance-based DHLI-BrA.

	Discrimination	Difficulty	Outfit	Infit
Q22	1.827	−0.718	0.513	0.694
Q23	0.856	−1.530	0.846	0.938
Q24	1.233	−0.459	0.737	0.803
Q25	0.762	−0.719	0.885	0.911
Q26	0.710	0.611	0.894	0.914
Q27	0.886	−0.182	0.849	0.872
Q28	0.858	1.020	0.848	0.893

Note. Infit = Weighted Mean Square Fit; Outfit = Unweighted Mean Square Fit.

**Table 5:** Spearman correlations between DHLI-BrA self-reported and performance-based with sociodemographic aspects, digital health literacy, health literacy, and quality of life.

Variables	DHLI-BrA Self-Reported		DHLI-BrA Performance-Based	
	r	*p*	r	*p*
Adolescent’s age	0.267	<0.001	0.324	<0.001
Average family income	0.167	0.015	0.242	<0.001
Digital Health Literacy–eHEALS-BrA ^1^	0.452	<0.001	0.065	0.294
Health Literacy–NVS ^2^	0.078	0.215	0.472	<0.001
Health Literacy–REALMD-20 ^3^	0.192	0.002	0.445	<0.001
Quality of life–physical	0.199	0.001	−0.059	0.346
Quality of life–psychological aspects	0.213	0.001	−0.101	0.106
Quality of life–social relationships	0.055	0.383	−0.071	0.257
Quality of life–environment	0.183	0.033	0.054	0.392

^1^ eHEALS-BrA: Version for Brazilian adolescents of the eHealth Literacy Scale; ^2^ NVS: Newest Vital Sign; ^3^ REALMD-20: 20-item Rapid Estimate Adult Literacy in Medicine and Dentistry. The significance level adopted was 5% (*p* < 0.05).

**Table 6:** Comparison of the mean (±SD) DHLI-BrA self-reported and performance-based scores with self-assessed general health and skills to use the Internet, access media, and search behavior for health information on the Internet.

Variables		DHLI-BrA Self-Reported	*p*	DHLI-BrA Performance-Based	*p*
General health self-assessment	Very Good	3.14 (0.39)	0.051 *	3.90 (1.73)	0.319 *
	Good	3.01 (0.37)		4.32 (1.73)	
	Regular	2.97 (0.34)		4.04 (1.63)	
	Bad/Very Bad	2.72 (0.51)		3.70 (1.48)	
Self-assessment of Internet usage skills	Very Good	3.11 (0.34)	0.002 *	4.24 (1.78)	0.012 *
	Good	2.97 (0.35)		4.27 (1.69)	
	Bad	2.87 (0.48)		3.33 (1.41)	
Frequency of Internet use	Every day	3.02 (0.37)	0.433 *	4.22 (1.71)	0.022 *
	Almost all	3.04 (0.42)		3.50 (1.55)	
	Almost never	2.78 (0.23)		2.17 (1.15)	
Do you have mobile Internet?	Yes	3.09 (0.36)	0.002 *	4.24 (1.79)	0.026 *
	Sometimes	2.92 (0.36)		4.19 (1.66)	
	No	2.95 (0.40)		3.32 (1.21)	
Searched for a doctor/dentist	Yes	3.07 (0.35)	>0.001	4.16 (1.75)	0.705 ^#^
	No	2.83 (0.39)		4.11 (1.59)	
Search for health information on the Internet	Yes	3.03 (0.37)	0.243	4.26 (1.68)	0.001 ^#^
	No	2.91 (0.42)		2.98 (1.69)	
Schedule appointment	Yes	3.06 (0.38)	0.014	4.29 (1.72)	0.090 ^#^
	No	2.96 (0.36)		3.97 (1.70)	
Read health information	Yes	3.04 (0.37)	0.246	4.36 (1.66)	0.002 ^#^
	No	2.97 (0.37)		3.59 (1.76)	
Use health apps	Yes	3.07 (0.39)	0.086	4.44 (1.76)	0.040 ^#^
	No	2.99 (0.36)		3.99 (1.66)	
Search for symptoms online	Yes	3.01 (0.37)	0.728	4.24 (1.71)	0.046 ^#^
	No	3.03 (0.38)		3.74 (1.70)	
Post an evaluation/review about a medical treatment	Yes	3.08 (0.37)	0.402	2.77 (1.96)	<0.001 ^#^
	No	3.01 (0.37)		4.290 (1.63)	

^#^ Kruskal–Wallis test/Mann–Whitney *U* test. * The significance level adopted was 5% (*p* < 0.05).

## Data Availability

The raw data supporting the conclusions of this article will be made available by the authors on request.

## Supplementary Materials

The following supporting information can be downloaded at: https://www.mdpi.com/article/10.3390/ijerph21111458/s1, File S1 Digital Health Literacy Instrument—Versão Brasileira para adolescentes (DHLI-BrA).
