# Supplementary material for: Evaluation of the Psychometric Properties of the Digital Health Literacy Instrument (DHLI-BrA) for Use in Brazilian Adolescents
Source: Int J Environ Res Public Health. 2024 Oct 31;21(11):1458. doi: 10.3390/ijerph21111458 (PMC11594048; doi:10.3390/ijerph21111458)
Supplement: Supplementary file 1 [file ijerph-21-01458-s001.zip › ijerph-3209428-supplementary.pdf]

***Digital Health Literacy Instrument* – Versão Brasileira para adolescentes (DHLI-BrA)**

Gostaria de perguntar sobre sua experiência em utilizar a internet. Para cada questão marque a resposta que melhor reflete suas habilidades e experiência ao usar a internet para situações de saúde. **Não existem respostas certas ou erradas. Nossa intenção é conhecer a sua experiência.**

	Muito Fácil	Razoavelmente fácil	Razoavelmente difícil	Muito difícil
**1.** O quanto é fácil ou difícil para você **digitar palavras** em um computador, tablet ou celular?	☐	☐	☐	☐
**2.** O quanto é fácil ou difícil para você **usar o mouse ou a tela** (por exemplo, para apontar o cursor para o lugar correto ou clicar)?	☐	☐	☐	☐
**3.** O quanto é fácil ou difícil para você **usar os botões / links** nos sites?	☐	☐	☐	☐
**4.** Quando você busca informações de saúde na internet, o quanto é fácil ou difícil para você **escolher uma informação** entre todas as encontradas?	☐	☐	☐	☐
**5.** Quando você busca informações de saúde na internet, o quanto é fácil ou difícil para você **escolher palavras ou frases de busca** adequadas para encontrar a informação que você procura?	☐	☐	☐	☐
**6.** Quando você busca informações de saúde na internet, o quanto é fácil ou difícil para você **encontrar a informação exata** que você procura?	☐	☐	☐	☐
**7.** Quando você busca informações de saúde na internet, o quanto é fácil ou difícil para você **decidir se a informação é confiável ou não**?	☐	☐	☐	☐
**8.** Quando você busca informações de saúde na internet, o quanto é fácil ou difícil para você **decidir se a informação foi escrita com interesses comerciais**? (por exemplo, por pessoas tentando vender um produto?)	☐	☐	☐	☐
**9.** Quando você busca informações de saúde na internet, o quanto é fácil ou difícil para você **verificar diferentes sites** para ver se fornecem a mesma informação?	☐	☐	☐	☐
**10.** Quando você busca informações de saúde na internet, o quanto é fácil ou difícil para você **decidir se a informação que você encontrou serve para você**?	☐	☐	☐	☐
**11.** Quando você busca informações de saúde na internet, o quanto é fácil ou difícil para você **aplicar no seu dia-a-dia** a informação que você encontrou?	☐	☐	☐	☐
**12.** Quando você busca informações de saúde na internet, o quanto é fácil ou difícil para você **usar a informação que você encontrou para tomar decisões sobre sua saúde** (por exemplo, sobre alimentação, uso de medicamentos ou para decidir se precisa da opinião de um médico)?	☐	☐	☐	☐

	Nunca	Às vezes	Frequentemente	Quase sempre
**13.** Quando você busca informações de saúde na internet, com que frequência acontece de você **não conseguir se localizar em um site ou na internet**?	☐	☐	☐	☐
**14.** Quando você busca informações de saúde na internet, com que frequência acontece de você **não saber como retornar a uma página anterior**?	☐	☐	☐	☐
**15.** Quando você busca informações de saúde na internet, com que frequência acontece de você **clicar em alguma coisa e ver algo diferente** do que você esperava?	☐	☐	☐	☐

Quando você está digitando uma mensagem ou um comentário (por exemplo: para seu médico, em um site ou uma rede social como YouTube, Instagram, WhatsApp, Telegram, Twitter, etc)....

	Muito Fácil	Razoavelmente fácil	Razoavelmente difícil	Muito difícil
**16.** o quanto é fácil ou difícil para você **escrever de forma clara** sua pergunta ou preocupação relacionada à saúde?	☐	☐	☐	☐
**17.** o quanto é fácil ou difícil para você **expressar sua opinião**, **pensamentos ou sentimentos por escrito**?	☐	☐	☐	☐
**18.** o quanto é fácil ou difícil para você **escrever sua mensagem** para que as pessoas entendam exatamente o que você quer dizer?	☐	☐	☐	☐

Você deve responder as questões abaixo (19,20,21) somente se já tiver postado uma mensagem em redes sociais (por exemplo: YouTube, Instagram ou Twitter, grupo de discussão aberto ou site de avaliação)**.**

	Nunca	Às vezes	Frequentemente	Quase sempre
**19.** Quando você publica uma mensagem online em uma rede social ou um grupo de discussão aberto, com que frequência você **acha difícil saber quem poderá ver**?	☐	☐	☐	☐
**20.** Quando você publica uma mensagem online em uma rede social ou um grupo de discussão aberto, com que frequência você **compartilha (com ou sem intenção) suas informações pessoais** (por exemplo: nome, endereço, localização, informação da escola, etc)?	☐	☐	☐	☐
**21.** Quando você publica uma mensagem online em uma rede social ou um grupo de discussão aberto, com que frequência você **compartilha (com ou sem intenção) informações particulares de outras pessoas** (por exemplo: nome, endereço, localização, informação da escola, etc)?	☐	☐	☐	☐

Questões para computador

22. Abaixo (figura 1), você vê parte da página inicial do site www.gov.br/saude/pt-br, um site nacional que fornece **informações oficiais do Ministério da Saúde**, como as campanhas de vacinação. Se você fosse **diminuir** essa página para abrir outro programa no seu computador, qual botão você usaria?

- ☐ Botão 1 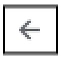
- ☐ Botão 2 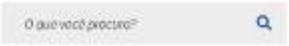

- ☐ Botão 3 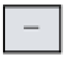
- ☐ Botão 4 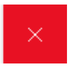
- ☐ Não sei

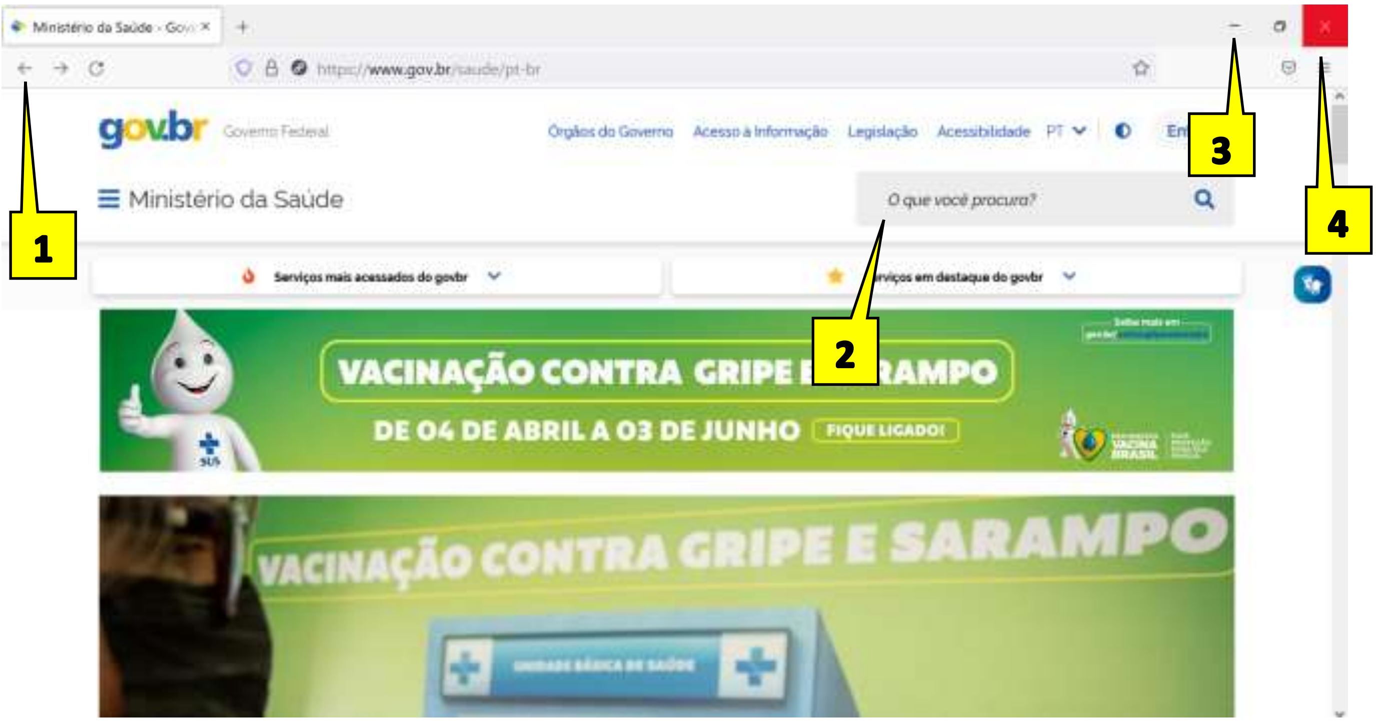

(Figura 1)

23. Imagine que você está buscando informações sobre a campanha de vacinação da gripe. Por meio do Google, você encontra o site www.gov.br/saude/pt-br (Veja figura 2). Agora, **você quer sair do site e voltar para seus resultados de pesquisa no Google**. Qual botão você usaria?

- ☐ Botão 1 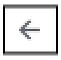
- ☐ Botão 2 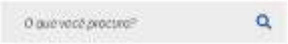

- ☐ Botão 3 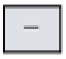
- ☐ Botão 4 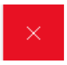
- ☐ Não sei

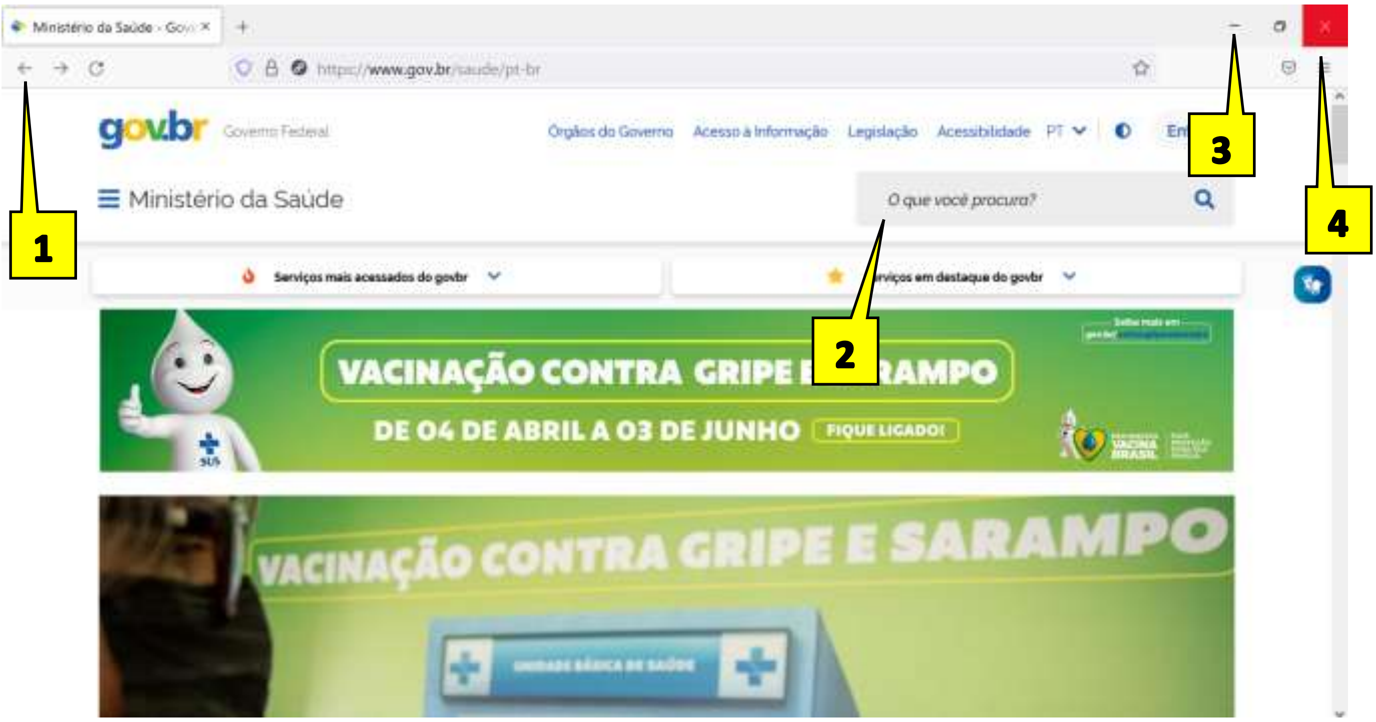

(Figura 2)

24. Imagine que você está em tratamento para diabetes. Você visita o site da **Sociedade Brasileira de Diabetes (SBD)** (veja figura 3). Você navega pelo site. **Qual tipo de informação você espera encontrar ao clicar no botão A**?

- ☐ Informação sobre diabetes
- ☐ Informação sobre o seu tratamento pessoal (como identificar qual a sua doença e medicamentos)
- ☐ Informação sobre complicações do diabetes
- ☐ Informação sobre a Sociedade Brasileira de Diabetes (SBD)
- ☐ Não sei

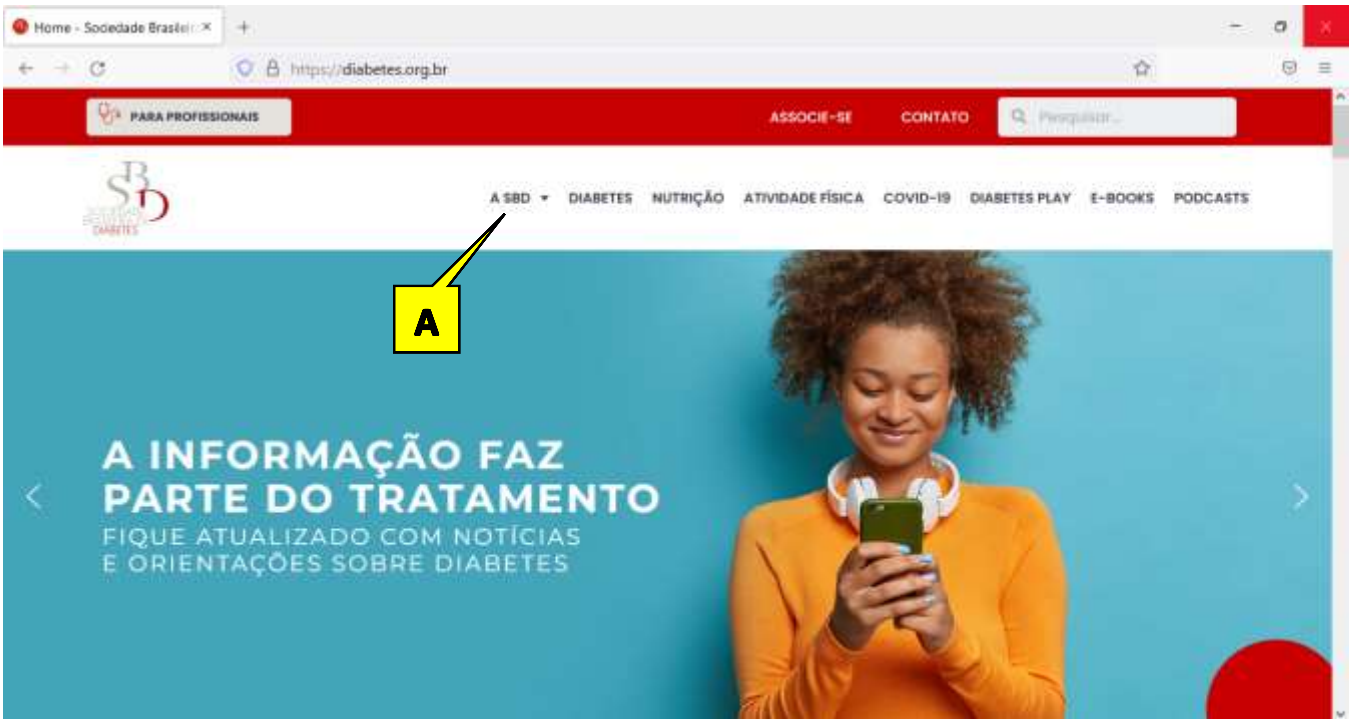

(Figura 3)

25. Imagine que você está procurando informações sobre cravos e espinhas. Você encontra o site abaixo, da **Sociedade Brasileira de Dermatologia (SBD)**, especialidade médica que trata as doenças de pele (veja figura 4). Você quer saber **quem é responsável pela Sociedade**. Em qual botão você clicaria?

- ☐ Botão 1: "A SBD"
- ☐ Botão 2: "Eventos"
- ☐ Botão 3: "Associe-se"
- ☐ Botão 4: "Contato"
- ☐ Não sei

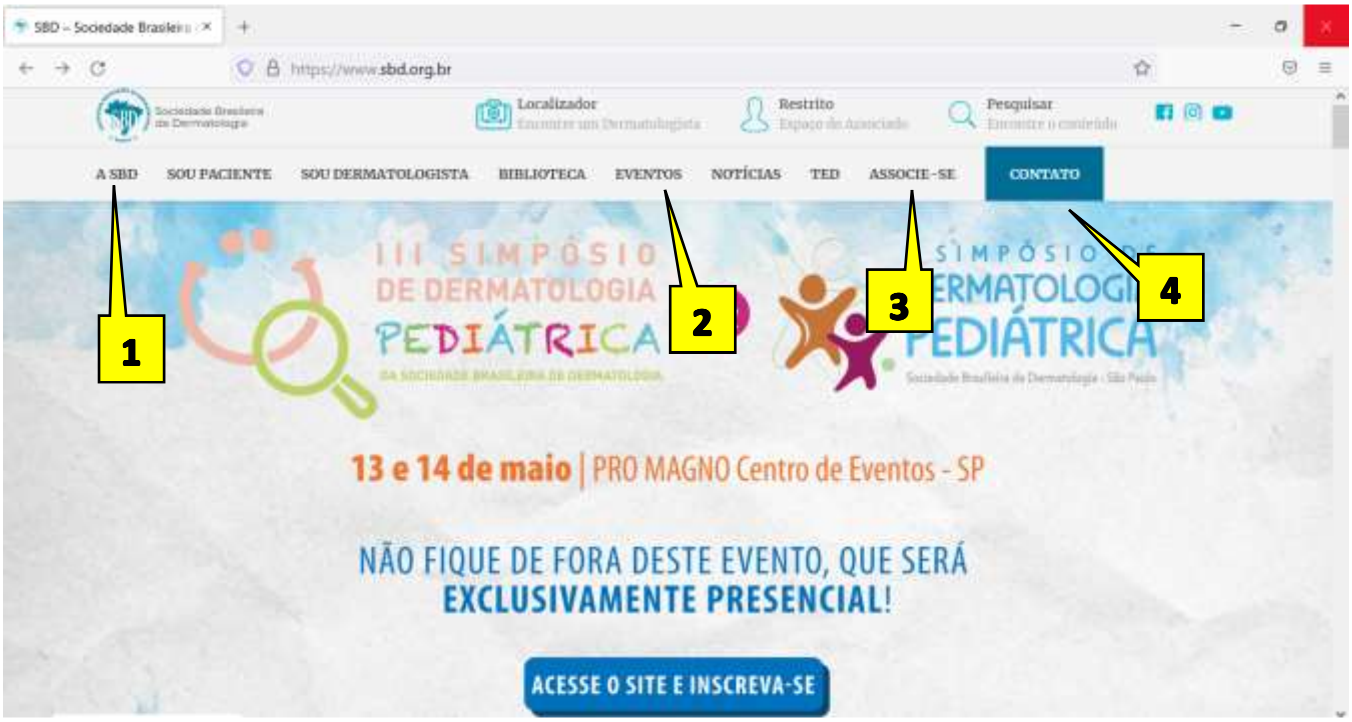

(Figura 4)

26. Imagine que você está muito cansado ultimamente. Sua amiga sugeriu que você poderia ter anemia. Você quer descobrir os sintomas exatos da anemia. Uma pesquisa via Google apresenta os resultados abaixo. Qual desses resultados provavelmente lhe daria uma **resposta mais correta e confiável**?

☐ Resultado de busca 1:

www.minhavida.com.br › saúde › tudo-sobre ▾

Anemia: o que é, tipos, sintomas e tratamentos | Minha Vida

A **anemia** indica hemoglobina baixa no sangue e nem sempre é falta de ferro na alimentação. Conheça os tipos, as causas e os **sintomas** de **anemia**.

☐ Resultado de busca 2:

bvsms.saude.gov.br › bvs › dicas › 69anemia ▾

Anemia - BVS - Ministério da Saúde - Dicas em Saúde

Sintomas: Os sinais e **sintomas** da carência de ferro são inespecíficos, necessitando-se de exames laboratoriais (sangue) para que seja confirmado o diagnóstico ...

☐ Resultado de busca 3:

www.drogarialiviero.com.br › blog › anemia-veja-quais-sao-os-sinto... ▾

Anemia: Veja quais são os principais sintomas e como tratar

Anemia - Os **sintomas** de **anemia** são provocados pela deficiência de glóbulos vermelhos no sangue, causada pela carência de nutrientes essenciais ao ...

☐ Resultado de busca 4:

forum.outerspace.com.br › Fóruns › Offtopic › Vale Tudo ▾

[MBML] Descobri que tenho ANEMIA | Fórum Outer Space - O único com ...

24 de nov. de 2008 - 35 postagens - 23 autores

Depois de muitos desmaios, perder muitos kgs, vomitar pra kct, descobri que estou com essa doença chamada **anemia**. Pelo o que eu entendi ...

☐ Não sei

27. Abaixo você encontra três mensagens de pacientes ou seus familiares em uma página de discussão da internet. Qual dessas mensagens **considera adequada** a própria privacidade e a privacidade dos outros?

- ☐ Mensagem 1 (da Aline)
- ☐ Mensagem 2 (da Luiza15)
- ☐ Mensagem 3 (da Juliana)
- ☐ Não sei

Re: Como estou...
De Aline >> Sábado, 16 de Out, 2021, 19:32

Oi,

Eu também moro no Bairro do Limão e usei as injeções de heparina e parei. Ainda tenho as injeções de Clexane; elas não me incomodam e funcionam bem para mim. Espero que funcione bem para você também e não o deixe doente. Te desejo ótimas férias.
Abraços, Aline

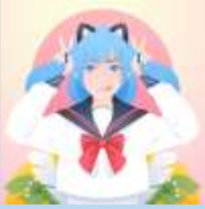
*

Aline Damasceno Costa
Mensagens: 452
Cidade: São Paulo
Tipo de trombofilia: Trombofilia da gravidez

Re: Meu pai precisa de quimioterapia...
De Luiza15 >> Sábado, 14 de Ago, 2021, 15:15

Olá Maria,

Obrigada por compartilhar suas experiências nesse momento difícil. Como está seu pai? Ele também tem dor nos ossos? Posso perguntar quantos anos ele tem? (Talvez você já tenha mencionado em uma das suas mensagens e eu não vi). Meu pai tem metástase nos ossos e uma forma agressiva de câncer de próstata. Desde dezembro ele está piorando. Agora ele está começando a ter dor nos ossos. Hoje vamos ao hospital Souza Costa às 17h, e minha mãe vai perguntar o que pode ser feito a respeito disso. Espero que a quimioterapia do seu pai funcione. Desejo força!

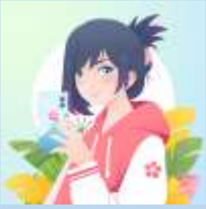
*

Luiza15
Mensagens: 3
Cidade: Rio de Janeiro
Registrada: sábado 14/agosto/2021 15:15

Alguém que tenha experiência com terapia ocupacional?...
De Juliana >> Sábado, 9 de Abr, 2022, 19:32

Olá a todos,

Meu irmão de 13 anos tem Transtorno do Déficit de Atenção com Hiperatividade (TDAH) desde quando era bebê. Ele tomou Ritalina por um tempo, mas meus pais estão considerando colocá-lo na terapia ocupacional. A terapia está disponível na nossa cidade, mas é muito cara. Alguém tem experiência com isso?
Obrigada, Juliana

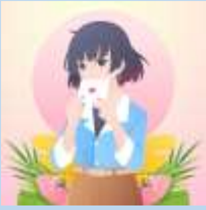
*

Juliana
Mensagens: 2
Registrada: sábado 09/abril/2022 19:32

* Imagens de domínio público By Pikisuperstar – br.freepik. https://br.freepik.com/fotos-vetores-gratis/cute-girl

28. Imagine que você vai viajar de férias para a Colômbia daqui a duas semanas. De repente você percebe que não sabe se você precisa de alguma vacina para essa viagem. Você decide escrever uma mensagem perguntando isso para seu médico.

Escreva abaixo o que você perguntaria ao médico nesta situação.

………...

…………………………..…………………………………………………………………………………

………...

…………………………………………………………..………………………………………………………

………...

…………………………………………………………..………………………………………………………

………...

Questões para celular - Android

22. Abaixo (figura 1), você vê a tela do celular com parte da página inicial do site www.gov.br/saude/pt-br, um site nacional que fornece **informações oficiais do Ministério da Saúde**, como as campanhas de vacinação. Se você fosse **diminuir** essa página para abrir outro aplicativo do seu celular, qual botão você usaria?

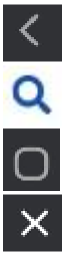

- ☐ Botão 1
- ☐ Botão 2
- ☐ Botão 3
- ☐ Botão 4
- ☐ Não sei

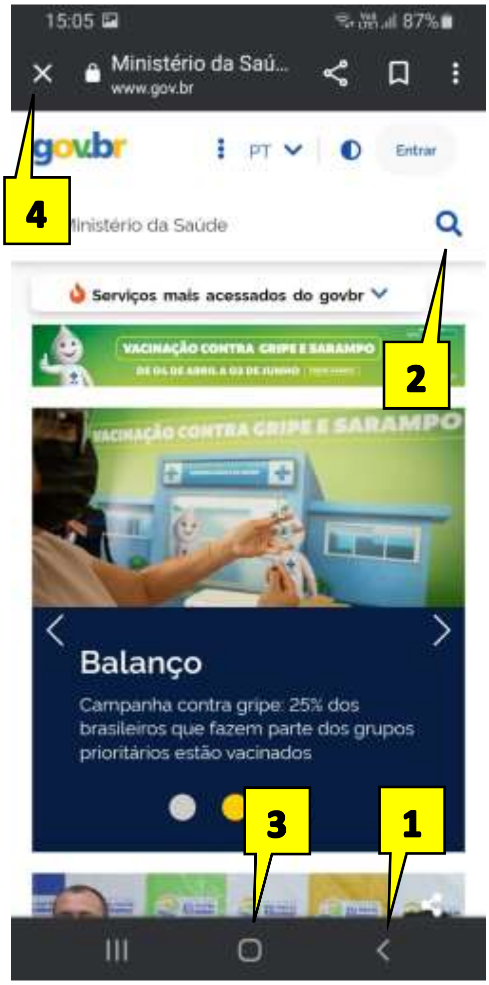

(Figura 1)

23. Imagine que você está usando seu celular para buscar informações sobre a campanha de vacinação da gripe. Por meio do Google, você encontra o site www.gov.br/saude/pt-br (Veja figura 2). Agora, **você quer sair do site e voltar para seus resultados de pesquisa no Google**. Qual botão você usaria?

- ☐ Botão 1 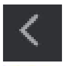
- ☐ Botão 2 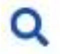
- ☐ Botão 3 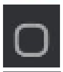
- ☐ Botão 4 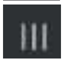
- ☐ Não sei

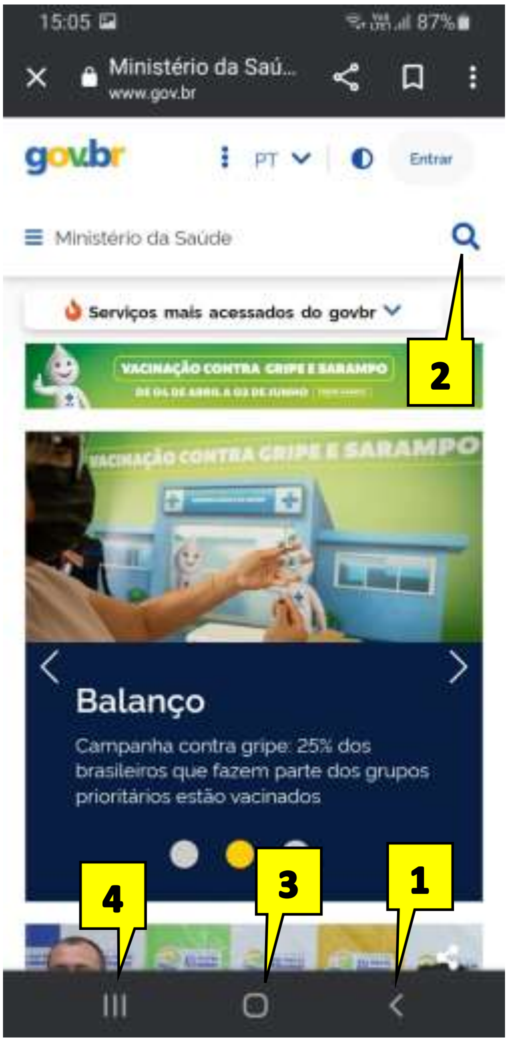

(Figura 2)

24. Imagine que você está em tratamento para diabetes. Você usa seu celular para visitar o site da **Sociedade Brasileira de Diabetes (SBD)** (veja figura 3). Você navega pelo site e clica no menu. **Qual tipo de informação você espera encontrar ao clicar no botão A**?

☐ Informação sobre diabete

☐ Informação sobre o seu tratamento pessoal (como identificar a qual sua doença e medicamentos)

☐ Informação sobre complicações do diabetes

☐ Informação sobre a Sociedade Brasileira de Diabetes (SBD)

☐ Não sei

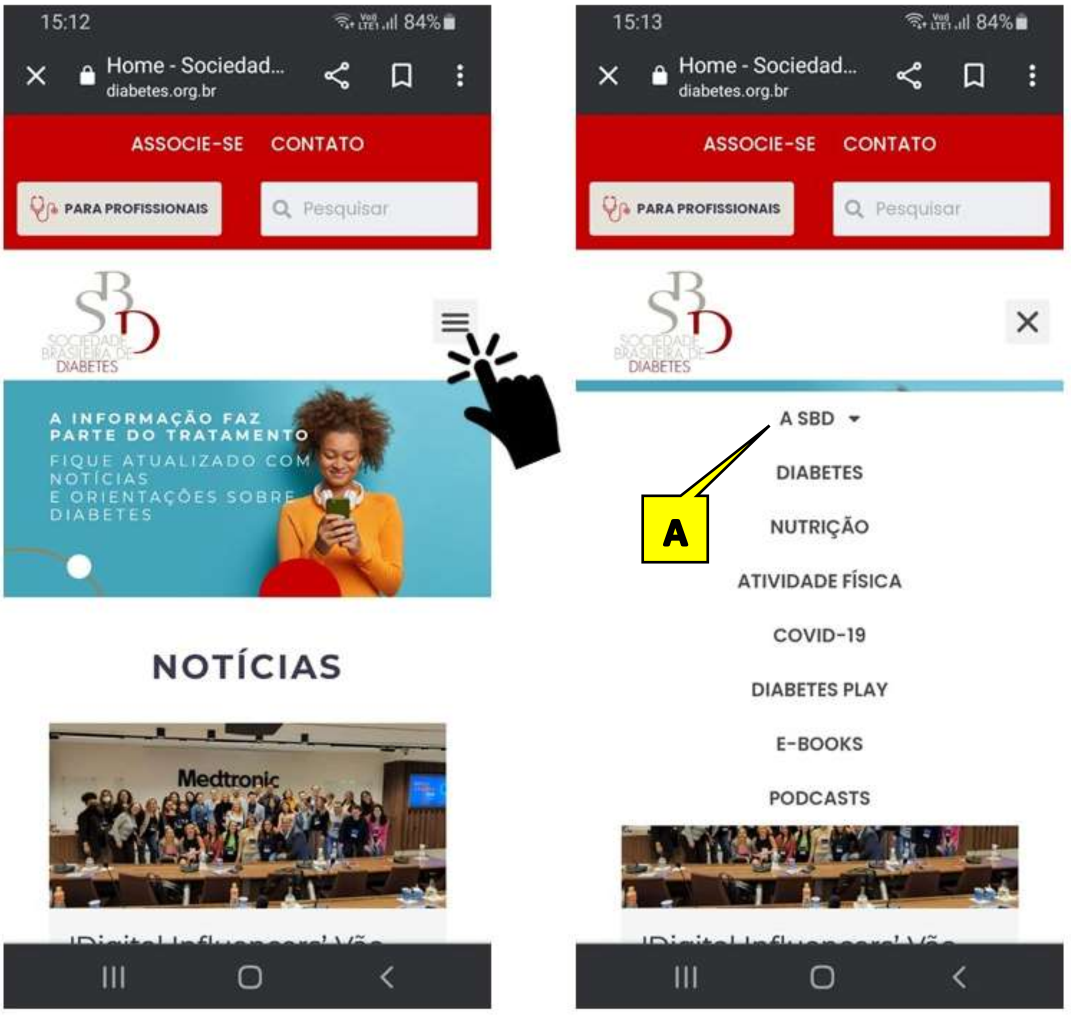

(Figura 3)

25. Imagine que você está procurando informações sobre cravos e espinhas. Você encontra o site abaixo, da **Sociedade Brasileira de Dermatologia (SBD),** especialidade médica que trata as doenças de pele (figura 4). Ao navegar pelo site, você clica no menu e quer saber **quem é responsável pela Sociedade**. Em qual botão você clicaria?

☐ Botão 1: “A SBD”

☐ Botão 2: “Eventos”

☐ Botão 3: “Associe-se”

☐ Botão 4: “Notícias”

☐ Não sei

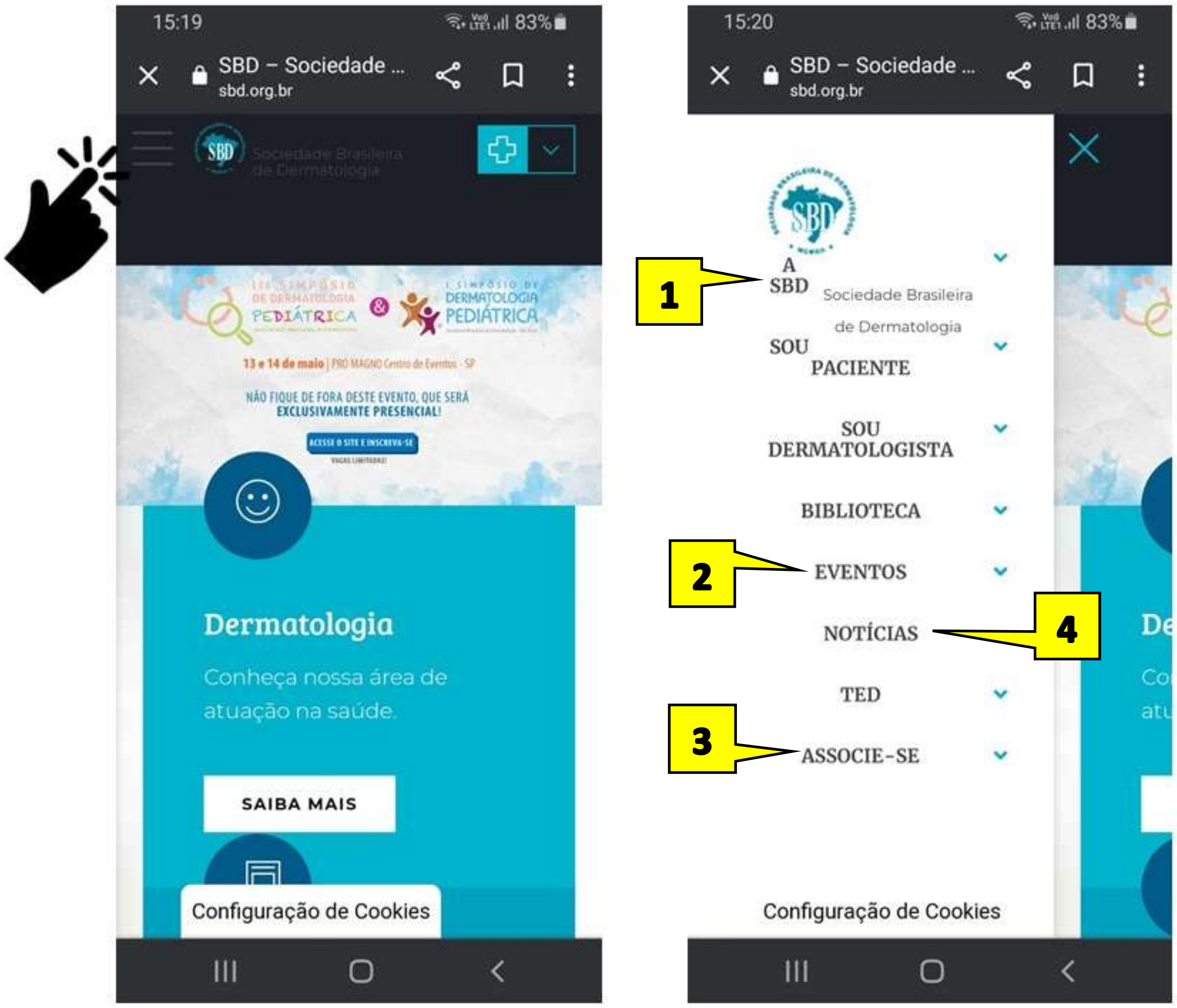

(Figura 4)

26. Imagine que você está muito cansado ultimamente. Sua amiga sugeriu que você poderia ter anemia. Você quer descobrir os sintomas exatos da anemia. Uma pesquisa com seu celular via Google apresenta os resultados abaixo. Qual desses resultados provavelmente lhe daria uma resposta **mais correta e confiável**?

☐ Resultado de busca 1:

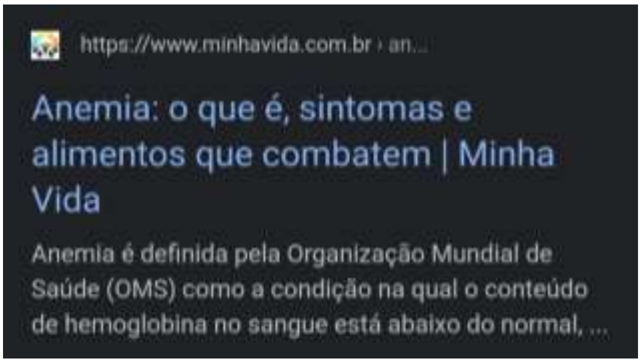

☐ Resultado de busca 2:

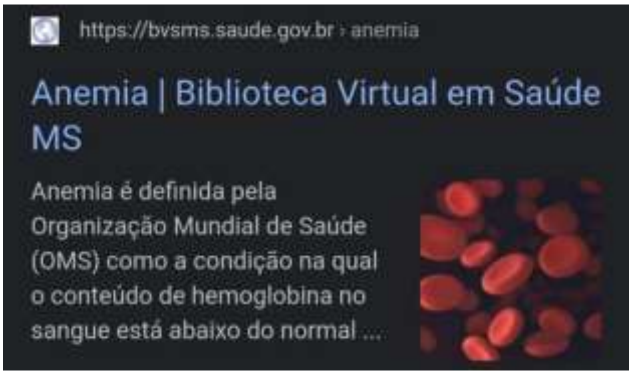

☐ Resultado de busca 3:

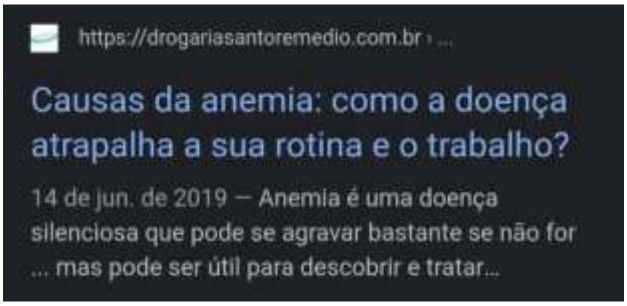

☐ Resultado de busca 4:

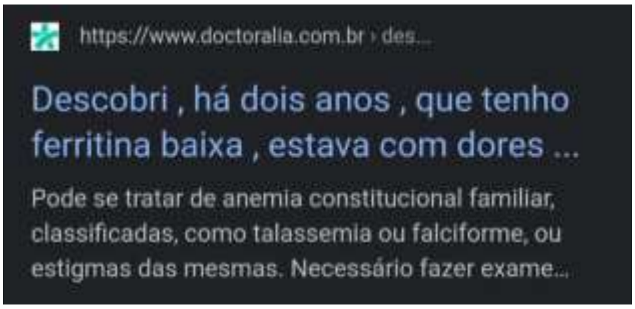

☐ Não sei

27. Abaixo você encontra três mensagens de pacientes ou seus familiares em uma página de discussão da internet. Qual dessas mensagens **considera adequada** a própria privacidade e a privacidade dos outros?

- ☐ Mensagem 1 (da Aline)
- ☐ Mensagem 2 (da Luiza15)
- ☐ Mensagem 3 (da Juliana)
- ☐ Não sei

Re: Como estou...
De Aline >> Sábado, 16 de Out, 2021, 19:32

Oi,

Eu também moro no Bairro do Limão e usei as injeções de heparina e parei. Ainda tenho as injeções de Clexane; elas não me incomodam e funcionam bem para mim. Espero que funcione bem para você também e não o deixe doente. Te desejo ótimas férias.
Abraços, Aline

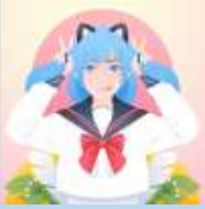 *

Aline Damasceno Costa
Mensagens: 452
Cidade: São Paulo
Tipo de trombofilia:
Trombofilia da gravidez

Re: Meu pai precisa de quimioterapia...
De Luiza15 >> Sábado, 14 de Ago, 2021, 15:15

Olá Maria,

Obrigada por compartilhar suas experiências nesse momento difícil. Como está seu pai? Ele também tem dor nos ossos? Posso perguntar quantos anos ele tem? (Talvez você já tenha mencionado em uma das suas mensagens e eu não vi). Meu pai tem metástase nos ossos e uma forma agressiva de câncer de próstata. Desde dezembro ele está piorando. Agora ele está começando a ter dor nos ossos. Hoje vamos ao hospital Souza Costa às 17h, e minha mãe vai perguntar o que pode ser feito a respeito disso. Espero que a quimioterapia do seu pai funcione. Desejo força!

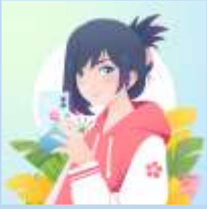 *

Luiza15
Mensagens: 3
Cidade: Rio de Janeiro
Registrada: sábado
14/agosto/2021 15:15

Alguém que tenha experiência com terapia ocupacional?...
De Juliana >> Sábado, 9 de Abr, 2022, 19:32

Olá a todos,

Meu irmão de 13 anos tem Transtorno do Déficit de Atenção com Hiperatividade (TDAH) desde quando era bebê. Ele tomou Ritalina por um tempo, mas meus pais estão considerando colocá-lo na terapia ocupacional. A terapia está disponível na nossa cidade, mas é muito cara. Alguém tem experiência com isso?
Obrigada, Juliana

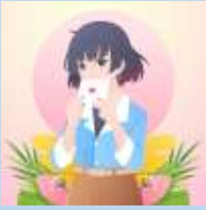 *

Juliana
Mensagens: 2
Registrada: sábado
09/abril/2022 19:32

* Imagens de domínio público By Pikisuperstar – br.freepik. https://br.freepik.com/fotos-vetores-gratis/cute-girl

28. Imagine que você vai viajar de férias para a Colômbia daqui a duas semanas. De repente você percebe que não sabe se você precisa de alguma vacina para essa viagem. Você decide escrever uma mensagem perguntando isso para seu médico.

Escreva abaixo o que você perguntaria ao médico nesta situação.

……...

……………………………..……………………………………………………………………………………….

……...

…………………………………………………………….………………………………………………………….

……...

…………………………………………………………….………………………………………………………….

……...

Digital Health Literacy Instrument – Versão Brasileira para adolescentes (DHLI-BrA)

Questões para celular - iOS

22. Abaixo (figura 1), você vê a tela do celular com parte da página inicial do site www.gov.br/saude/pt-br, um site nacional que fornece **informações oficiais do Ministério da Saúde**, como as campanhas de vacinação. Se você fosse **diminuir** essa página para abrir outro aplicativo do seu celular, qual botão você usaria?

☐ Botão 1 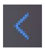

☐ Botão 2 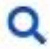

☐ Botão 3 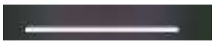

☐ Botão 4 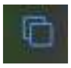

☐ Não sei

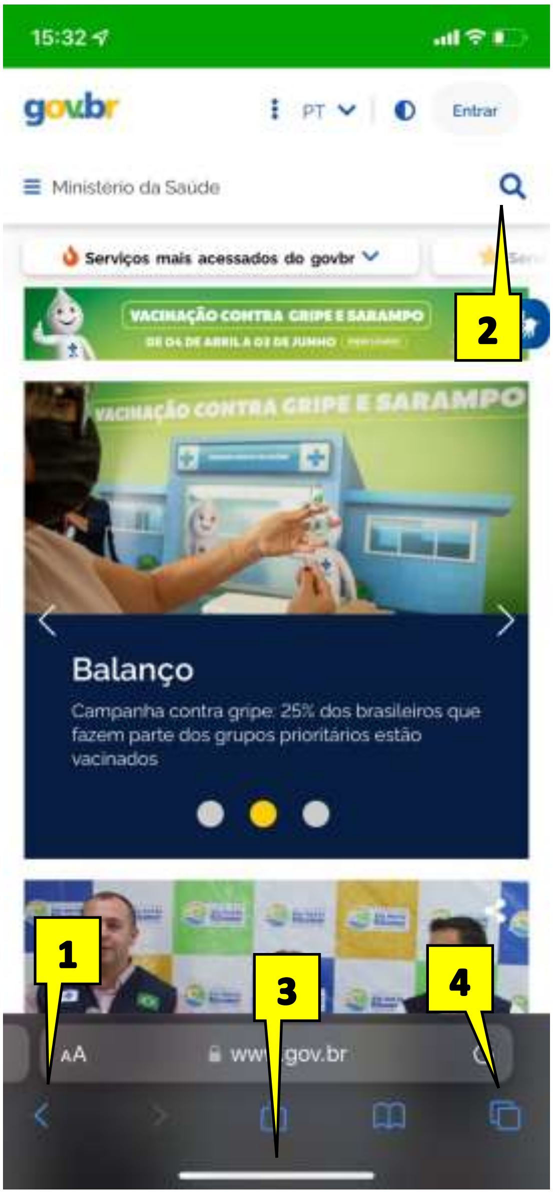

(Figura 1)

23. Imagine que você está usando seu celular para buscar informações sobre a campanha de vacinação da gripe. Por meio do Google, você encontra o site www.gov.br/saude/pt-br (Veja figura 2). Agora, **você quer sair do site e voltar para seus resultados de pesquisa no Google**. Qual botão você usaria?

- ☐ Botão 1 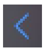
- ☐ Botão 2 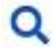
- ☐ Botão 3 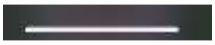
- ☐ Botão 4 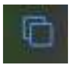
- ☐ Não sei

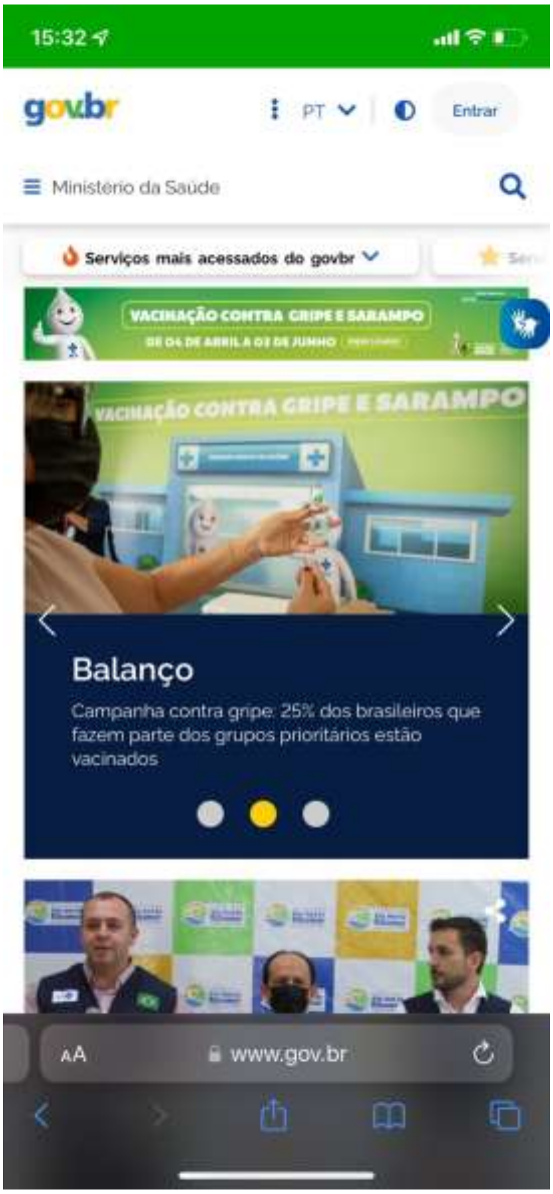

(Figura 2)

24. Imagine que você está em tratamento para diabetes. Você usa seu celular para visitar o site da **Sociedade Brasileira de Diabetes (SBD)** (Veja figura 3). Você navega pelo site e clica no menu. **Qual tipo de informação você espera encontrar ao clicar no botão A**?

☐ Informação sobre diabete

☐ Informação sobre o seu tratamento pessoal (como identificar a qual sua doença e medicamentos)

☐ Informação sobre complicações do diabetes

☐ Informação sobre a Sociedade Brasileira de Diabetes (SBD)

☐ Não sei

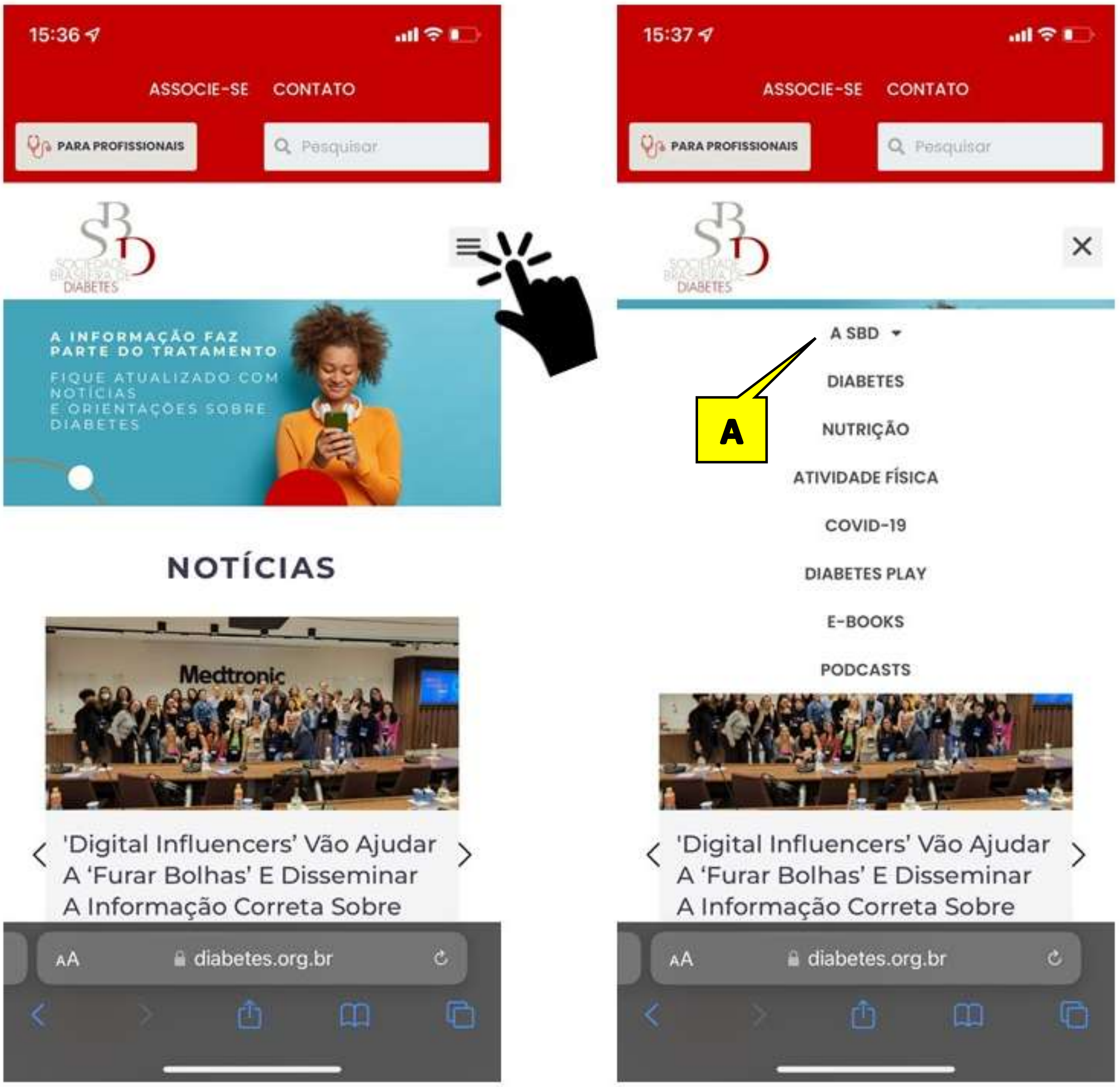

(Figura 3)

25. Imagine que você está procurando informações sobre cravos e espinhas. Você encontra o site abaixo, da **Sociedade Brasileira de Dermatologia (SBD),** especialidade médica que trata as doenças de pele (figura 4). Ao navegar pelo site, você clica no menu e quer saber **quem é responsável pela Sociedade**. Em qual botão você clicaria?

☐ Botão 1: "A SBD"

☐ Botão 2: "Eventos"

☐ Botão 3: "Associe-se"

☐ Botão 4: "Notícias"

☐ Não sei

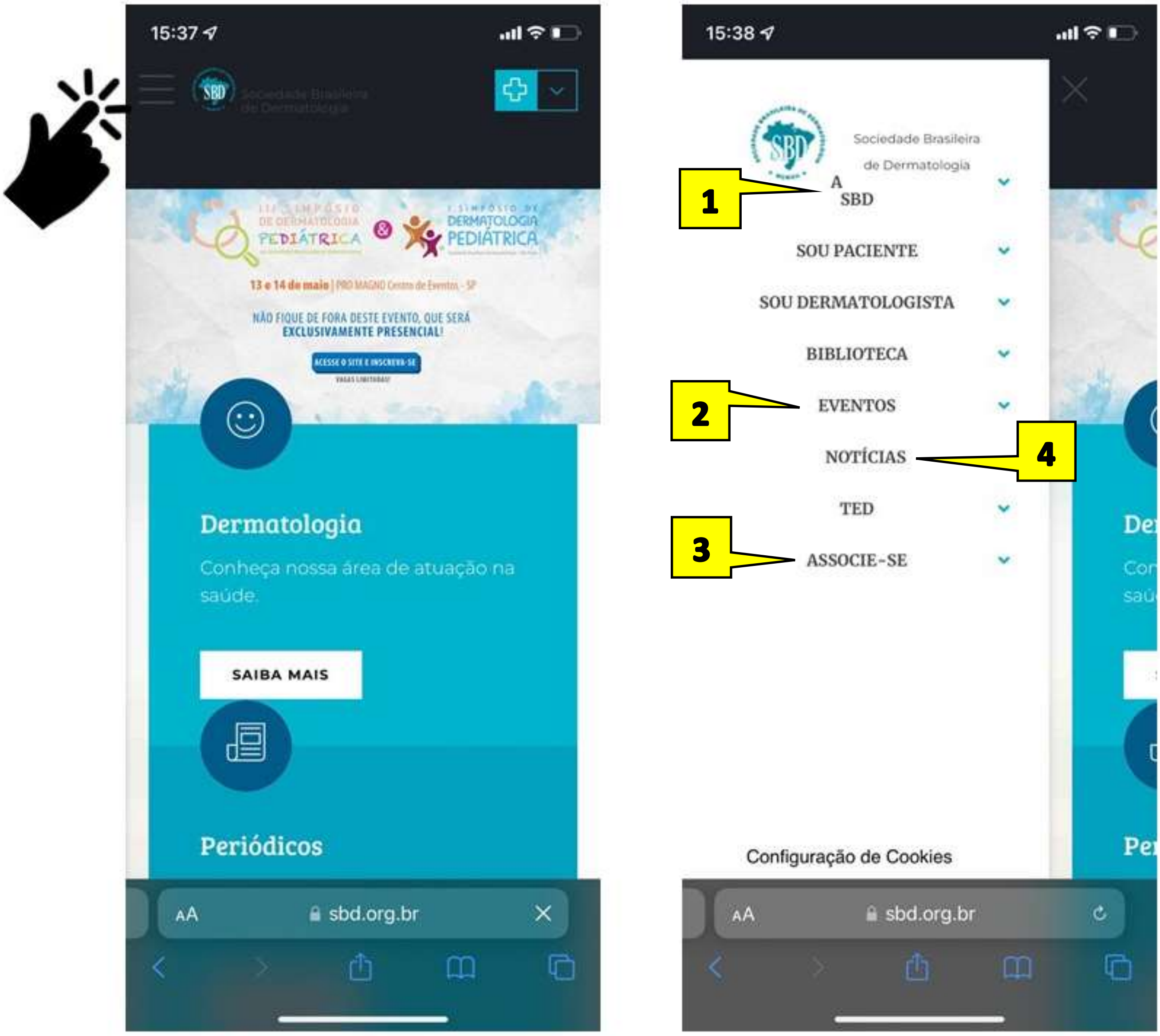

(Figura 4)

26. Imagine que você está muito cansado ultimamente. Sua amiga sugeriu que você poderia ter anemia. Você quer descobrir os sintomas exatos da anemia. Uma pesquisa com seu celular via Google apresenta os resultados abaixo. Qual desses resultados provavelmente lhe daria uma resposta **mais correta e confiável**?

☐ Resultado de busca 1:

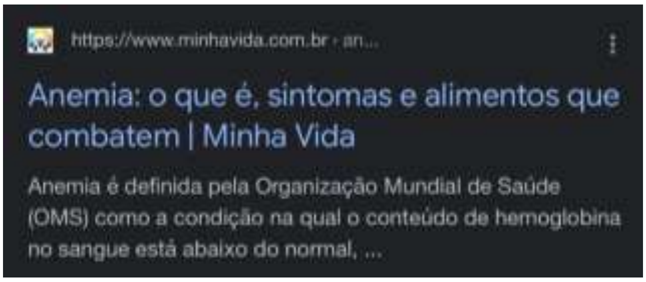

☐ Resultado de busca 2:

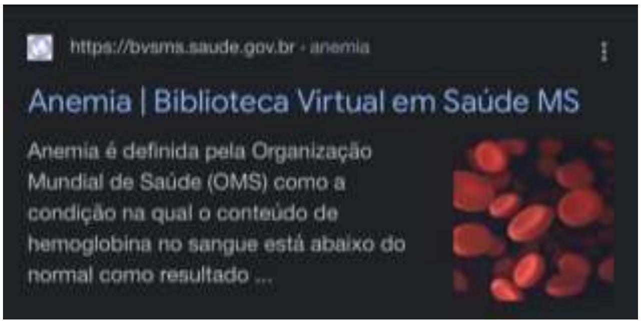

☐ Resultado de busca 3:

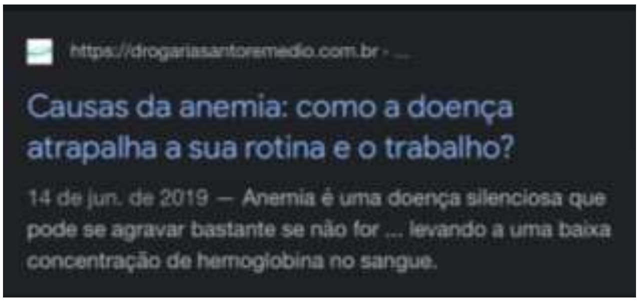

☐ Resultado de busca 4:

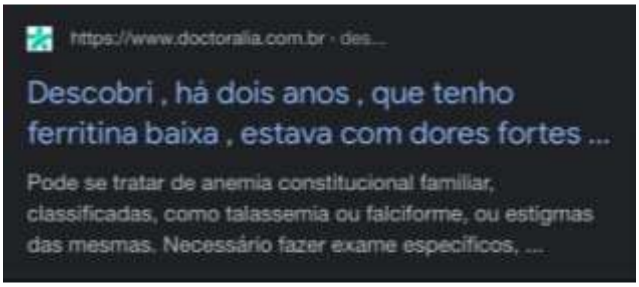

☐ Não sei

27. Abaixo você encontra três mensagens de pacientes ou seus familiares em uma página de discussão da internet. Qual dessas mensagens **considera adequada** a própria privacidade e a privacidade dos outros?

- ☐ Mensagem 1 (da Aline)
- ☐ Mensagem 2 (da Luiza15)
- ☐ Mensagem 3 (da Juliana)
- ☐ Não sei

Re: Como estou...
De Aline >> Sábado, 16 de Out, 2021, 19:32

Oi,

Eu também moro no Bairro do Limão e usei as injeções de heparina e parei. Ainda tenho as injeções de Clexane; elas não me incomodam e funcionam bem para mim. Espero que funcione bem para você também e não o deixe doente. Te desejo ótimas férias.
Abraços, Aline

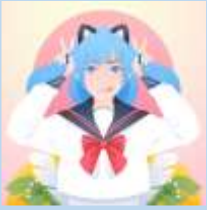
*
Aline Damasceno Costa
Mensagens: 452
Cidade: São Paulo
Tipo de trombofilia: Trombofilia da gravidez

Re: Meu pai precisa de quimioterapia...
De Luiza15 >> Sábado, 14 de Ago, 2021, 15:15

Olá Maria,

Obrigada por compartilhar suas experiências nesse momento difícil. Como está seu pai? Ele também tem dor nos ossos? Posso perguntar quantos anos ele tem? (Talvez você já tenha mencionado em uma das suas mensagens e eu não vi). Meu pai tem metástase nos ossos e uma forma agressiva de câncer de próstata. Desde dezembro ele está piorando. Agora ele está começando a ter dor nos ossos. Hoje vamos ao hospital Souza Costa às 17h, e minha mãe vai perguntar o que pode ser feito a respeito disso. Espero que a quimioterapia do seu pai funcione. Desejo força!

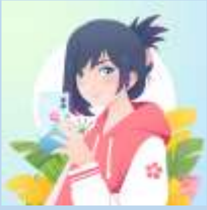
*
Luiza15
Mensagens: 3
Cidade: Rio de Janeiro
Registrada: sábado 14/agosto/2021 15:15

Alguém que tenha experiência com terapia ocupacional?...
De Juliana >> Sábado, 9 de Abr, 2022, 19:32

Olá a todos,

Meu irmão de 13 anos tem Transtorno do Déficit de Atenção com Hiperatividade (TDAH) desde quando era bebê. Ele tomou Ritalina por um tempo, mas meus pais estão considerando colocá-lo na terapia ocupacional. A terapia está disponível na nossa cidade, mas é muito cara. Alguém tem experiência com isso?
Obrigada, Juliana

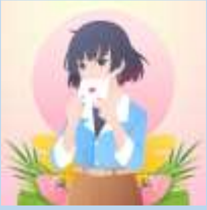
*
Juliana
Mensagens: 2
Registrada: sábado 09/abril/2022 19:32

* Imagens de domínio público By Pikisuperstar – br.freepik: https://br.freepik.com/fotos-vetores-gratis/cute-girl

28. Imagine que você vai viajar de férias para a Colômbia daqui a duas semanas. De repente você percebe que não sabe se você precisa de alguma vacina para essa viagem. Você decide escrever uma mensagem perguntando isso para seu médico.

Escreva abaixo o que você perguntaria ao médico nesta situação.

……...

……………………………..…………………………………………………………………………………….

……...

…………………………………………………………..………………………………………………………….

……...

…………………………………………………………..………………………………………………………….

……...
